# What Is the Purpose in Retiring?

**DOI:** 10.1093/geroni/igaa057.1430

**Published:** 2020-12-16

**Authors:** Rachel Best, Patrick Hill

**Affiliations:** Washington University in St. Louis, Saint Louis, Missouri, United States

## Abstract

Sense of purpose is related to many positive physical and mental health outcomes (Boyle, Barnes, Buchman & Bennett, 2009). Unfortunately, sense of purpose declines as adults age and particularly with retirement (Pinquart, 2002). Retirement is often measured dichotomously, however, many people are retiring gradually and do not fit clearly as retired or not retired. Moreover, people retire for different reasons ranging from seemingly non-negative reasons (financial incentives or wanting to spend more time on non-work activities) to more negative reasons (physical health problems, mental health problems, cognitive decline, pressure to retire, or getting fired). The current study (N = 558; mean age = 60.17) used data from a Hawaiian sample in which participants completed self-report surveys on retirement status and activity engagement, as well as a measure of purpose (Scheier et al., 2006). We examined whether the role of retirement on sense of purpose differed by form of or motive for retirement. Results indicated the fully retired population reported significantly lower sense of purpose than both the still working and the partially retired populations, but these two groups did not differ. Participants who retired for negative reasons reported significantly lower sense of purpose than those who retired for non-negative reasons. Greater activity engagement was significantly related to sense of purpose for all retirement statuses. However, this relationship was stronger for the partially retired population. Future research should examine further the role of partial retirement for maintaining sense of purpose in older adulthood.

